# On the Presence of Air in the Veins as a Cause of Death

**Published:** 1864-03

**Authors:** Jas. Sumner Greene

**Affiliations:** Dorchester, Mass.


### ON THE PRESENCE OF AIR IN THE VEINS AS A CAUSE OF DEATH.

By JAS. SUMNER GREENE, M. D.,

*Of Dorchester, Mass.*

[Continued from February number.]

To return to the case under consideration. At the autopsy there was found to be slight but general peritonitis. (We have no account of the previous health of the patient.) The uterus contained a fœtus of from four and a half to five months, with membranes intact. No marks of mechanical injury were noticed. The placenta was easily to be broken down, and there were two small clots beneath it. No signs of hemorrhage were found in the vagina nor about the clothes nor bed. Dr. Hitchcock, hearing the husband's testimony, formed the opinion that she died from forcible entrance of air into the circulation, through the uterine sinuses. His opinion being made known, the body was disinterred and re-examined five days after death. It was intended to tie all the vessels and open the heart under water; but one of the ligatures slipped off when the vessel was cut, and a puff of air was heard by all present. There was engorgement, probably cadaveric, of the large viscera.

(A careful report of this case, by Dr. Hitchcock, will be laid before the American Medical Association at its next meeting.)

Enough evidence has now been advanced of the liability to the entrance of air into the veins during certain operations in surgery, and under certain conditions of the uterine system, to put every practitioner on his guard against the circumsances which have been shown to be dangerous. Indeed, those most skeptical as to the cases cited admit the prudence of proper precautionary measures. But are there not other occasions, of more or less frequent occurrence, in which the possibility of danger is almost equally manifest as in some of these?

If death has been produced by the mere puncture of the seton needle in the neck, in the hands of a skillful operator, what shall we say of the danger in the operation of tracheotomy, now so frequently practised? Here we meet veins of at least equal calibre with those wounded in certain of the fatal cases, and which the most careful surgeon cannot always avoid. These veins lie on, or within, the borders of the region shown to be the most dangerous, and at the time of such operation are generally turgid with blood. What more is needed than the entrance of the scalpel at a certain angle, combined with traction of the tissues in a certain direction, to open the door to the entrance of the unwelcome visitor? Yet we are instructed by surgeons, if a vein is wounded, to complete the operation as soon as possible, without reference to bleeding, as its completion will at once relieve the loaded vessels and check the hemorrhage; while no word of warning is concerning the possibility of this fatal complication.

Even the simple insertion of a subcutaneous syringe may be conceived to carry with it possible danger. These instruments are liable to take up and eject as much air as water, as we know from experience. The improved syringes of glass show its presence, while those in ordinary use, made of gutta-percha, conceal it.

The dangers to be guarded against in those more rare but very important operations, the transfusion of blood in anæmia, and the injection of water impregnated with salts, in cases of cholera, cannot fail to suggest themselves here. Indeed, there is a statement by the late Dr. Francis, of N. Y., (*Med. Magazine*, Nov. 1832), that in the few autopsies of bodies after venous injection, during the cholera epidemic, great cerebral congestion was found, and air within the heart, aorta, and larger bloodvessels. Allusion was made at the same time to the horrors of death after this operation, but we find no account of symptoms. (See also Experiments on the Transf. of Blood, by Dr. Jas. Blundell, *Medico-Chirurg Trans.*, ix. p. 65.)

We have quoted cases of the introduction of air into the bloodvessels by means of suicidal wounds; but neither Guthrie nor any other of the great authorities in military surgery have noted this as a complication of wounds received on the battle-field. One allusion of this sort we do find. It is stated by Sir Charles Bell (*Practical Essays*, Part I,) that Baron Larrey, while examining with him some sketches of the wounded at the battle of Waterloo, noticed particularly the case of a young man who had been wounded in the lower part of the neck. "Well I know," said this excellent surgeon, "how that young man must have died. I have seen so many wounded during my campaigns, and die from air drawn into the veins." In sabre wounds about the dangerous region we should suppose this accident might be of frequent occurrence; less so in gunshot wounds, where retraction is more likely to close the mouths of the vessels; yet even here it is easy to conceive that in many cases they may remain patulous until closed by coagula. This is a subject which well deserves investigation, and the opportunities offered by the present war to settle it should not be overlooked. The difficulty of making sufficiently careful autopsies near the battle-field, where, if at all, these accidents must take place, is an objection which might be obviated by the detailing of a surgeon for that especial purpose.

We come now to consider certain possibilities more important even than those just recited; more important because they come within the range of every physician's daily practice. We have seen that the injection of gas or liquids into the uterine cavity is attended with danger; yet we have no doubt that both agents are still used for procuring uterine contractions, for the relief of pain, or as disinfectants, without the slightest precaution.

It is not long since Prof. Simpson has discontinued and discouraged the actual injection of carbonic acid gas into the os uteri. (*Edinburgh Med. Journ.*, Sept. 1861, and *American Journ. Med. Sci.*, Jan. 1862, p. 271.) Two instances of sudden death during such injection are reported by Scanzoni. The one of which we have read the particulars (*Brit. and For. Med.-Chir. Rev.*, vol. xxvi, p. 274,) seems to us to point very clearly towards entrance of the gas into the veins as the cause of death.

Mr. James, of the City Lying-in Hospital, London, passes an elastic catheter through the os, between the uterine walls and the membranes, to the extent of four or five inches, and injects cold water by means of an elastic bottle. (Simpson, *loc. cit.*) We can hardly conceive of a more dangerous practice, at least in the hands of those unaccustomed to it, when we remember the ease with which a vein might be punctured and air admitted with the water, to say nothing of the injurious effects of water itself in the circulation. Prof. Simpson himself was greatly alarmed, on one occasion, at seeing a patient faint under the use of Higginson's syringe, an effect which, he suggests, was probably due to some of the fluid (p rhaps air) getting into the circulation. Dr. Robt. Barnes (*Obstet. Trans.* 1861, p. 119) states that several cases are known in which death has speedily followed the injection of fluid into the non-pregnant uterus, and says that unless care is taken, air is very apt to be thrown up with the water by any of the ordinary siphons or pumping syringes He refers to a case reported by Dr. Guiller (*Gaz. Méd.*, 1857), in which injections of water being ordered to cleanse the vagina of a woman wearing a pessary, water mingled with air was thrown into the uterus and forced through the Fallopian tubes into the peritoneal cavity, adding that it may be conjectured that air also found its way into the bloodvessels. Dr. H. R. Storer, of Boston, goes still farther and condemns—as we think very justly—the use of Keiler's caoutchouc air pessaries, on the same ground. In an able paper recently read by him before the Suffolk District Medical Society on artificial dilatation of the cervix uteri (*Boston Medical and Surgical Journal*, July 3, 1863), he states that several cases of death from the use of these means have occurred, and names the entrance of air into the vascular system as the most probable explanation of this fatality. Dr. Storer informs us that he has always opposed the use of any of the means by which this class of accidents is rendered possible, and he considers it one of the chief merits of his method of dilatation, that it places the occurrence of danger from this source out of the question.

If it is urged that accident under such circumstances as we have alleged must be so rare as not to deserve notice, since scarcely any are recorded, we point in answer to the fact that 50 years ago the possibility of accident from this cause under *any* circumstances was not even suspected, while the first instance of the kind, occurring after parturition in the human species, was not noticed until several years after Legallois and Ollivier had suggested its possibility. If we are forewarned of the danger, it may serve not only to bring so light proofs of its reality which would otherwise have slept, but to preserve valuable lives which would otherwise be sacrificed; and it should be remembered that a single accident must weigh more in the affirmative than scores, or even hundreds, of fortunate cases could do in the negative.

Before proceeding to consider the mechanical conditions favoring the accident, and its proximate cause, some mention should be made of a class of cases very analogous to the preceding, viz., those in which gas seems to be developed within the vessels instead of admitted from without. In fact, there appears to be a regular gradation of instances, from such as are evidently wholly physiological to such as are as clearly due to the operation of physical laws; so that the two classes merge into each other, and it is difficult to decide to which some of the cases belong. Thus, the fact of the secretion of gas by mucous lining of the stomach and intestines is too common to require comment. Instances are reported on good authority where the skin and bladder have exhibited the same phenomena. Sir Francis Smith witnessed an instance of this sort, in a hypochondriac patient, where the water of the bath in which he lay became covered with bubbles, which proceeded from the surface of his body. (*Dub. Journ. of Medical Sciences*, 1841, p. 454.) Prof. Puchelt thinks that in certain persons bubbles of air not unfrequently form and explode within the current of the circulation itself, and is familiar with one kind of palpitation, which gives the precise idea of bubbles bursting in the heart. (Jones and Sieveking, *Path. Anat.*, p. 364.) A step further brings us to those cases wherein sudden death has occurred, and where careful dissection, frequently made so early after death as to leave no room for changes of decomposition, has brought to light nothing but the presence of air in the heart and veins to explain the event. Cases of this sort are mentioned by Morgagni (*op cit.*, secs. 20, 24) as having been remarked by Verdriesius, Grætzius, Ruysch, and himself; and the death of Albrechtus, Prof. of Anatomy at Gottingen, is cited as being of the same kind. Dr. Cless, of Stuttgard, has published 14 cases, in most of which the patients were suffering from blood-poisoning. (G. May, *Trans. of Path. Soc. of London*, vol. ix, p. 154.) Air is said to have been found in the veins, after death from strychnia poisoning, from hydrophobia, and from inhalation of chloroform. (*Id.*) Mr. May reports several cases. In one, the patient being taken ill, the medium vein was opened and much frothy blood escaped; sudden death occurred, and air was found in the heart. In another, there was ulceration of the cæcum, and air was found in the veins, from the ulcer to the heart. A third occurred during the catamenial period, and air was traced from the left lateral ligament of the uterus to the heart. In a case of gangrene of the great toe, air was found from the seat of the lesion to the heart. In a 5th case, the patient recovered from two attacks of sudden cough, followed by insensibility, lividity, coldness of extremities, rapid pulse and respiration, ending with the expectoration of much frothy mucus tinged with blood, and the third attack proving fatal, the autopsy disclosed air in the pulmonary vessels and frothy blood in the cavities and vessels of the heart. In all these cases the autopsy was performed within 30 hours after death, and there was no evidence of decomposition.

It is impossible not to associate this class of cases with that immediately under discussion; and we must admit the possibility of some peculiar and unfrequent condition of the system, which predisposes to the reception of the noxious agent, or to the more rapid development of fatal effects. This of course is mere conjecture, but if we admit its possibility, it will help to explain the supposed rareness of the accident, compared with the frequency of the occasions in which the mechanical conditions favoring it are presented. In those cases where gas is unquestionably developed within the vessels, it would be useful to its exact composition, and thus determine whether it is generated by decomposition of the blood, or merely set free from solution in the fluid. Prof. Bacon informs us that investigations have yet to be made to decide this question, and that the necessary experiments and analyses, though complicated, would be entirely practicable.

If the gas is really set free from solution, it is probably in consequence of some change in the blood, as incompatible with life as actual putrefactive change; for though it is demonstrated by the experiments of Bischoff, Magnus, J. Davy, and others, that a large percentage of carbonic acid and oxygen gas both exist in healthy blood (Carpenter's *Prin. Human Physiol.*, p. 1598, and Lehmann's *Physiol. Chemistry*, vol. i, p. 570, and vol. ii, p. 474), yet it is unlikely that either of these could be liberated from solution, and thus cause death mechanically, without some essential change in the vital properties of that fluid which would render it incapable of fulfilling its part in the economy. Yet the mechanical cause may, nevertheless, and probably does, have its share in the fatal issue, and perhaps controls the character of the symptoms.

The mechanical conditions under which air has obtained entrance into the circulation during surgical operations have been pretty thoroughly canvassed. It was shown by Messrs. Barry and Poisseuille that a marked influence was exerted on the current of blood, in certain of the larger veins at the summit of the thorax, by the movements of the chest in respiration, its flow being accelerated during inspiration, and checked, and to a certain degree reversed, during expiration, producing the phenomenon of a venous pulse in that region. This was shown to be due to a sucking action, exerted upon the venous current by the expanding chest. Within this region, the *espace dangereuse* of Amussat, including the lower portion of the internal jugular, the subclavian, and the upper half of the axillary, the opening of a vein was shown to be always followed by the admission of air. It was noticed by M. Bouillaud that when the movements of respiration became feeble, the flux and reflux in the veins no longer correspond to the movements of the thorax, but became synchronous with the beat of the heart; and we have noticed this change in the frequency of the venous pulse, in the exposed jugular vein of a cat, before any vessel had been punctured. It corresponds entirely to the circumstances already noticed in connection with the lapping sound produced by the entrance of air at a wound. M. Berard demonstrated by careful dissection, that those portions of veins, subject to flux and reflux, were united to neighboring bones and muscles by aponeurotic laminæ or bridles which kept them expanded, while elsewhere nothing hindered their natural tendency to collapse from atmospheric pressure when not distended by blood. (Velpeau, *op cit.*, p. 453.) Velpeau and others denied the possibility of the accident beyond the range of the venous pulse, but it has been sufficiently demonstrated by various observers, as well as by the experiments of the commission, that many circumstances may and do operate to extend the area of danger, so as to include smaller veins, as well as those at a greater distance from the heart, and the limits of the danger have not yet been definitely fixed. Any circumstance which prevents the collapse of the vessel under the pressure of the atmosphere invites the entrance of air. Among these conditions are a forced inspiration, during which the platysma, sterno-cleido-mastoid and anterior portion of the trapezoid muscles are lifted off the veins which underlie them, thus allowing them to dilate (Sir C. Bell, *Practical Essays*, p. 17); dragging the forcible distension of a vein during an operation, either by the necessary position of the patient, as in a case of Dr. Warren, or by manipulation of the parts while dissecting, as in the case of Dupuytren and Roux; adhesion of the vein to morbid growths; and thickening of its coats from chronic inflammation. Something depends on the position of the patient, whether erect or recumbent, the former being more favorable to the occurrence of the accident from the comparative emptiness of the veins about the neck, thus placed above the horizontal plane of the heart. The character of the incision into the vein also has an important bearing on the chances of accident. Thus, if a portion of its wall is removed, yet not enough to prevent the onward flow of blood within it towards the heart, air is likely to be drawn with it into the circulation; if the vein is partially divided trasversely at the bottom of a wound, the drawing apart of the flaps may furnish an opening sufficiently large, while complete division transversely would favor immediate collapse and retraction if the veins were not canalized in any of the ways before mentioned. A fact stated by Sir Charles Bell helps to explain the ready entrance of air into a vein through which the current of blood continues to flow. "When water flows through a tube, the tube being gradually larger at its further extremity, and a lesser tube inserted into it, water will not flow from the larger tube into the smaller, but from the smaller into the larger. This corresponds with the course of the blood in the veins; for the lesser veins are inserted into a series of trunks, gradually enlarging in their diameters till they reach the heart. In these circumstances, a hole in the side of the tube will not discharge water, but will admit air." (*Op. cit.*)

The conditions by which air enters by the uterine veins have been less carefully studied. Prof. Simpson says (Reid, *op. cit.*, p. 580), "As to the mechanism of the introduction of air in such cases, I think we can understand it, when we remember that the interior of the uterus after delivery, especially opposite the late seat of the placenta, is studded with venous orifices, and that, if air does once become introduced into the uterine cavity, from relaxation of the walls of the organ, it will be liable to be forced into these orifices, and hence into the general venous circulation; providing the uterus, in again contracting, is unable to expel its contents through the os uteri." Dr. McClintock refers to the large size of the veins of the gravel womb, their freedom from inosculation, the total absence of valves, and their termination on the internal surface of the uterus, at the site of the placenta, by large open orifices. "If the uterus," he remarks, "be examined soon after delivery at full term, the majority of these apertures will readily admit a goose-quill, and some will even allow the little finger to penetrate without laceration. During contraction of the uterus, all the openings are hermetically closed; but when it is relaxed, they again become proportionately more or less petulous. From this it is manifest, that the same condition of the organ which causes flooding, is exactly that which is indispensable for the ingress of air; so that the latter, when it does take place, is almost of necessity preceded, or accompanied by hemorrhage. (Churchill's *Midwivery*, p. 529.) This is far too sweeping a statement. It evidently contemplates the possibility of the accident only as a sequence of parturition; yet even here we can readily conceive of the possibility of air obtaining entrance into the uterine veins during the progress of the normal contractions, when little or no blood need escape. Indeed, we must conclude with Simpson, that it is by means of these very contractions that, in many cases, air is driven into the vessels. Facts corroborate this view; for we see by the cases reported, that hemorrhage is by no means a constant accompaniment of the accident, and that where it has occurred, it has apparently been far from profuse. This coincides with the remark of Wattman, and with the experience of the commission, that want of bleeding from the opened vein is a prominent sign of the accident. To the question, How comes the air into the uterine cavity? Dr. Churchill answers: "It may penetrate, doubtless, during the process of expulsion of the child, or during the interval before the expulsion of the placenta, or it may be the result of decomposition. That air is expelled from the uterus occasionally, during or immediately after labor, we know; so that we may conclude with Dr. Cormack: 'I have not only no difficulty in believing, but am constrained to admit, that should any impediment be offered, in such cases, to the free exit of air by the os uteri, it must be forced into the uterine veins, were their mouths not protected by coagula.' " (*Id.*) M. Amussat thinks that the phenomena of the introduction of air into the uterine veins may be explained by the same mechanism as for the dangerous region, *i. e.*, by the respiratory movements which are transmitted even to the uterus by the flux and reflux of the intestines; and hence if the uterus does not contract, and if the vessels of its walls remain patulous, we can conceive that aspiration of air may take place as at the neck, though doubtless less easily. (*Op Cit.*, p. 245.)

The proximate cause of death has been a subject of much controversy, and it has not yet been explained beyond a doubt. Morgagni states, and appears to accept, the opinion of most of the earlier physiologists, that death occurs from the great distension of the walls of the heart, which was likened to over-distension of the bladder with urine; though he suggests the possibility of apoplexy, in some cases, from the effect of air in the vessels of the brain. (*Op. cit.*, secs. 23, 24.) Bichat also believes that the air becomes mortal on arriving at the brain, but how it operates upon this organ to produce death, he considers not worth investigating. "*Qu'importe le comment?*" he asks, "*le fait seul nous interesse.*" (*Op. cit.*) Nysten reasserted the opinion of the earlier experimenters, that death, in the rapidly fatal cases, was due to over-distension of the right cavities of the heart, which he believed occasioned such a dragging of the fibres of the left ventricle as to oppose the free exercise of its contractility; though, in those cases where the fatal issue was delayed, he acknowledged that its cause was in the lungs. (*Op. cit.*, p. 166.) Magendie, Dupuytren, and others accepted this view. (MERCIER, *Gaz. Méd.*, 1837, p. 485.) MM. Piedagnel and Leroy thought that the air forced into the lungs by the contractions of the right ventricle, and suddenly expanded by the change of temperature, ruptured the pulmonary capillaries, and produced emphysema, and thus stoppage of the circulation. (MERCIER, *op. cit.*, 1837, p. 484.) M. Ollivier accepted this theory as probable in the more prolonged cases, but preferred that of Nysten for the instances where death was sudden, suggesting the probable rarefaction of air in the heart as the occasion of over-distension. (*Op. cit.*, p. 71.) M. Amussat seems to have held a similar opinion. (*Op. cit.*, p. 240.) M. Marechal de Calvi thought that carbonic acid was disengaged from the blood by the oxygen of the air mixed with it, and that this operated as a poison upon the heart. (ERICHSEN, *op. cit.*, p. 4.)

M. Mercier names, 1st, stasis of the venous blood, from the inability of the heart to propel so elastic a fluid as air; 2d, interruption of the arterial circulation from the same cause, combined with the frothiness of the blood in the pulmonary capillaries; while, 3dly, the pressure of air in the arterial system may give the last blow to the organs already enfeebled by the privation of their natural excitant. (*Gaz. Méd.*, p. 485, 1837.)

M. Denot assigns as the cause, accumulation of air in the right cavities of the heart, consequent upon inability of the auriculo-ventricular valve to prevent the reflux of air from the ventricle into the auricle. (*Id.*, p. 728.) Sir Charles Bell believes that air interferes with the functions of animal life, by entering the vessels of the medulla oblongata. (*Op. cit.*) Dr. Wing asks: "Can we not suppose that the air impairs the power of circulation, 1st, by distending the heart with its own volume; and, 2dly, by causing an imperfect closure of the valves, and thus permitting a reflux of blood at each contraction of the ventricle, which causes, in its turn, an increased disturbance, until it goes beyond the power of reaction to overcome." (*Loc. cit.*)

Professor Wattman's theory is, progressive decrease in the activity of the heart, and of its influence upon the circulation, in consequence of the sudden over-distension of the venous system by the mixture of blood and air; the insufficient oxidation and decarbonization of the blood in the lungs, with simultaneous and equal decrease in, and impediment to, its electro-magnetic influence; the diminution of the exciting and nutritive qualities of the blood, and its consequent diminished capacity to maintain the function of those parts of the brain, spinal marrow, lungs, and heart, the united action of which are essential to life. (*Am. Jour. Med. Sci.*, vol. 9, N. S., p. 170.)

M. Bouillaud assigns a threefold cause. 1st. Distension of the right cavities of the heart, impeding its contractions. 2d. Spumous blood in the branches of the pulmonary artery preventing the free passage of blood through the lungs. 3d. Entrance of air into the vessels of the brain causing compression of that organ. (*Op. cit.*) Dr. Cormack attaches importance only to the first of these reasons, Reid to the first and second conjoined, while others, among whom are Poisseuille (*Gaz. Méd.*, 1837, p. 671), Erichsen (*op. cit.*, p. 15), Bernard (*Des

*Substances Toxiques et Médicamenteuses*, p. 163), and Dalton (*op. cit.*, p. 510), are content with the second of these alone.

We have thus gone over with the principal theories offered to account for the fatality attending the accident. Our limits will not permit us to discuss them, though there is not one which does not seem open to objections more or less weighty. Those referring death to the presence of air in the brain or spinal cord, or to emphysema of the lungs, are disproved by the result of dissection in a large majority of cases.

Against the theory of poisoning from the elimination of carbonic acid, are adduced the experiments of Nysten, which show that the injection of that gas is even less injurious than air, being more readily soluble. (*Op. cit.*, p. 81.) Against the theories indicating obstruction at the heart, it is urged that that organ is often seen contracting after the respiration has ceased, and that it is by no means always found distended. (*Erichsen*, p. 10.) Finally, it is objected against the theory of obstruction of the lungs by frothy blood, that the sudden injection of an equal volume of water produced the same effect as air. (*Br. and For. Med. Rev.*, vol. xxv., p. 462.) While we believe that the disturbances produced in the economy by the presence of the foreign element are as complex as are the relations of interdependence between the different organs and functions of the body, and cannot be satisfactorily explained in the present imperfect state of our knowledge of these relations, we admit that the weight of evidence indicates that one of the chief troubles is the impediment offered to the passage of blood through the lungs. It should be remarked, however, that the symptoms produced may be all referred to disordered nerve influence, so that whatever mechanical conditions combine to strike the blow, or on whatever link or series of links of the vital chain it falls first or chiefest, it must be felt by the cerebral centres before any response becomes manifest.

We are now prepared to give an opinion on the practical question referred to at the beginning, viz., as to the nature and amount of evidence necessary to prove that a given case of death was due to this cause; and we reply, in general terms, that a sudden death taking place under any of the circumstances shown to be favorable to the occurrence of this accident, and not adequately and satisfactorily accounted for in any other way, affords strong presumptive evidence in favor of this as the cause; that if in addition the case presents certain of the prominent symptoms which we have remarked, such as violent disturbance of the respiratory and cardiac movements, great prostration, or convulsive spasms, and especially if the hissing sound indicating the entrance of air, or the churning sound, significant of its presence in the heart, is heard at the same time, the fact may be considered as established without the additional evidence of a *post mortem* examination, which should nevertheless be always obtained, if possible, that the proof may be rendered unexceptionable, as well as for the scientific interest attaching to it. At the same time we cannot too strongly urge the importance of accurately noting the minutest details, as far as this can be done, without sparing every possible effort for the relief of the distressing symptoms. Above all, auscultation of the heart should never be omitted. In conducting an autopsy in a suspected case, the length of time which had elapsed since death should be noticed, together with the temperature, whether warm or cold, both of the body itself and of the atmosphere in which it had been kept, and the general condition of the body as to cadaveric changes. The calvarium should not be removed until after the heart is examined, and great care should be taken in opening the thorax not to wound any considerable vein. As a precaution against this, it would be better to saw the sternum through, than to disarticulate it from the clavicle. The contents of the heart, and of such of the larger vessels as give evidence of the presence of air, should be evacuated under water, notice being taken whether the air, if any issues, comes to the surface in a single wave, or in a multitude of minute bubbles. The former would indicate that the air was present, unmixed with blood; the latter that it was mixed with that fluid, forming a froth. We should not, however, with Prof. Dalton (*op. cit.*, p. 517), make this a decisive diagnostic sign, indicating whether the air gained entrance during life or not, unless the autopsy took place immediately after death, which, of course, it seldom can; for a short time might suffice for the froth to subside, and the air to be extricated entirely from the blood, which was doubtless mixed with it by the action of the heart. The gas might be readily collected in an inverted beaker, and measured approximately, and in a case admitting of doubt as to whether the gas was atmospheric air or not, the rough analysis resorted to by Roux would suffice. After collecting the gas, he decided that it was not ammonia because it was insoluble in water; it was not carbonic acid, for it gave no precipitate with lime-water; a lighted bougie plunged in it burned with the same activity as in the surrounding air, hence it was not nitrogen; neither was it oxygen, which would have given increased brilliancy to the flame, while hydrogen would have taken fire itself while extinguishing the taper. Thus, by the process of elimination he arrived at the conclusion that the gas found in the heart was atmospheric air. (MERCIER, *op. cit.*, p. 483.)

We now pass to a brief consideration of the remedial and preventive measures. As to the former the indications are—1st. To prevent further ingress of air. 2d. To remove from the heart and lungs, if possible, that already admitted. 3d. To sustain the vital organs in the performance of their functions.

The first indication can be promptly met in surgical cases; indeed Wattman places almost his whole reliance on the prompt application of the necessary measures for this end, asserting that, if attended to when the first signs of the accident are manifest, a fatal termination can always be avoided. When the lapping sound is heard, or any symptom pointing to the entrance of air is noticed, instantly desist from the operation and press the point of the index finger upon the spot whence the sound proceeds. Finish the operation without removing the finger. If the hand is in the way, let the finger of another hand be placed upon the vein between the wound and the heart, until the first finger is slid along the vein to the necessary distance. As soon as possible, permanent closure is to be obtained either by quickly uniting the lips of the wound, by ligature on the vein, by torsion, or by the lateral ligature, whichever of these methods seems most practicable, from the nature of the incision, the relation of the vessel to the surrounding parts, and its size and importance. The lateral ligature is claimed by Wattman as peculiar to himself, and we must refer the reader to his work, or the reviews already quoted for an account of it. Its application is to large veins wounded through only a portion of their diameter, and it is intended to close the orifice without obliterating the calibre of the vessel. Ferguson thinks there would be danger of exciting inflammation in the vein by this method, and that simple closure of the wound with a little pressure would be preferable. (FERGUSON's *Surgery*, p. 630.) The prompt application of compression, has apparently been the means of saving life in many of the cases reported, among others in one of those of Dr. Warren, and in the late one of Dr. Clark; but, of course it is inapplicable to cases where the uterus is the source of the accident, and it must be confessed that little can be done in those cases under the first indication. If a clot or other obstruction exist in the cervix uteri or vagina, it should be speedily removed.

Under the second indication, the most important measure is probably artificial respiration, which, by keeping up the action of the heart, may lead to the propulsion of the spumous blood through the pulmonary capillaries. Amussat and Blandin recommend suction by means of a tube passed through the wounded vein, or the right jugular, into the auricle, combining this with pressure on the chest. Magendie and Rouchoux advise suction alone, and Gerdy, simple compression. The objections to suction are the danger of admitting more air, and of injuring the inner walls of the vessels or heart, and we do not know that it has ever been practiced. M. Amussat ascribed recovery in the case already mentioned reported by him, to pressure which he employed upon the chest, at the same time leaving the opening free. The patient should be so placed that the opening in the vessel shall be directed upwards, and be on a somewhat higher level than the heart. The pressure should be made to alternate with relaxation, and care should be taken during the intervals to close the orifice of the vein.

To meet the third indication artificial respiration is also required, together with the administration of brandy and diffusible stimulants. Various other measures of more or less value have been recommended; among these the recumbent position and pressure on the abdominal aorta or iliac arteries, both of which means promote the afflux of arterial blood to the brain, and thus help to sustain it in the exercise of its functions. If there are signs of great venous congestion, bleeding at the right jugular vein, care being still taken to avoid the further entrance of air, may at once relieve the brain and the right side of the heart of excess of venous blood. For the rest, the usual remedies applied in ordinary syncope should be invoked, such as aspersion with cold water, the application of ammonia to the nostrils, and warmth to the extremities. Dr. Cormack recommends the alternate use of hot and cold douches combined with stimulants, in those cases which threaten asphyxia. "In many instances," he says, "repose, dashing cold water in the face, keeping the surface warm, and time may be the only means which ought to be used." (CHURCHILL, *op. cit.*, p. 531.) If the immediate danger is averted, care is to be taken against the supervention of inflammation in the lungs.

The preventive measures consist simply in avoiding as far as possible all the known conditions which favor its occurrence.

In most of the cases the means of avoidance suggest themselves at once in connection with the danger. A few measures require particular mention. Erichsen recommends with Gerdy enveloping the chest in tight flannel bandages to prevent the deep, gasping inspirations which favor the ingress of air. This has been objected to on the ground that respiration would be too much interfered with, while the admission of air would not be prevented; but aside from this, the now universal use of anæsthetics during surgical operations renders the respiration sufficiently regular by controlling the emotional influences which would otherwise disturb it. On the other hand compression of the hypogastrium after delivery, by means of a bandage around the abdomen, as advised by M. Amussat (*op. cit.*, p. 245), seems a judicious prophylactic measure. He also recommends that the thighs be kept approximated. M. Dupuytren counselled that, in the removal of tumors in the dangerous region, they should be divided into several portions by crucial incisions, and thus removed piecemeal, to avoid the traction necessary in dissecting them whole from the adjacent tissues, and M. Graedemur has taken a similar precaution since the occurrence of the accident under his hands. (*Am. Journ. Med. Sci.*, July, 1842.) M. Blandin also advocates this plan of crucial incisions, and in addition, in view of the fact that the accident seems to be more uniformly fatal when occurring towards the close of an operation wherein considerable blood has been lost, proposes that the extirpation of tumors be commenced at the part most exposed to the access of air, instead of leaving that until the last, as is usual. (*Gaz. Médicale*, 1843, p. 51.) Compression of the large veins between the seat of operation and the heart has been recommended, but would often be objectionable from causing turgidity of the vessels. For the rest, a full knowledge of the conditions favoring the entrance of air into the veins, and the keeping that knowledge in mind during all operations and in the care of all cases in which there is a possibility of danger; these are the best safeguards against it.

In the imperfect sketch which we have now given of this important subject, we have endeavored to give especial prominence to its practical aspect. We have not taken up single cases and analyzed the evidence for and against each; having neither space nor disposition to do this, but preferring to admit at the outset, that every case cited, while it adds something of value to the whole mass of evidence, is of itself imperfect; but we have presented an array of facts and authorities which taken as a whole, must, we think, convince any candid mind that fatal accidents have occurred, and may again occur, in all of the ways which those cases illustrate. By giving a *résumé* of the cases already known, with an account of the conditions under which they occurred, by indicating other circumstances under which there is more or less reason to apprehend danger, and by citing instances of an analogous class of cases which it is practically desirable to distinguish from these, we have hoped to aid the practitioner to deal intelligently with any case of the kind which he may have the misfortune to encounter, and therefore have been content to pass over very briefly the medico-legal and therapeutie aspects of the subject. In conclusion, we commend the matter to the attention of the practical men of the medical profession, whatever their specialty, feeling confident that it is well worth the trouble of further investigation,

